# Subcutaneous Administration of Tramadol after Elective Surgery Is as Effective as Intravenous Administration in Relieving Acute Pain and Inflammation in Dogs

**DOI:** 10.1100/2012/564939

**Published:** 2012-06-18

**Authors:** Salisu Buhari, Kalthum Hashim, Goh Yong Meng, Noordin Mohamed Mustapha, Siew Hua Gan

**Affiliations:** ^1^Department of Veterinary Clinical Studies, Faculty of Veterinary Medicine, Universiti Putra Malaysia, 43400 Serdang, Selangor, Malaysia; ^2^Department of Veterinary Surgery & Radiology, Faculty of Veterinary Medicine, Usmanu Danfodiyo University Sokoto, PMB 2346, Sokoto, Nigeria; ^3^Department of Veterinary Preclinical Studies, Faculty of Veterinary Medicine, Universiti Putra Malaysia, 43400 Serdang, Selangor, Malaysia; ^4^Department of Veterinary Pathology and Microbiology, Faculty of Veterinary Medicine, Universiti Putra Malaysia, 43400 Serdang, Selangor, Malaysia; ^5^Human Genome Centre, School of Medical Sciences, Universiti Sains Malaysia, Kelantan, 16150 Kubang Kerian, Malaysia

## Abstract

Subcutaneous (SC) administration of tramadol was compared with intravenous (IV) administration to evaluate analgesia following canine ovariohysterectomy (OHE). Healthy female dogs (*n* = 12) between 1 and 3 years of age (1.95 ± 0.65 years), weighing between 10.5 and 17.1 kg (13.12 ± 1.95 kg), were used. Pain was assessed at baseline before surgery and then hourly for 8 hr after surgery. Tramadol was administered both SC and IV at a dose of 3 mg/kg and provided significant postoperative analgesia, as indicated by analgesiometry, **β**-endorphin levels, and interleukin 6 (IL-6) levels. The respiratory rates and rectal temperatures remained normal and were not significantly different between or within the groups. A significant increase in heart rate was observed at 4 hr for dogs in both groups relative to the baseline, but there was no significant difference in heart rates between the groups at any time point. A significant decrease in mechanical pain threshold was observed within each group after surgery, but both groups responded similarly, suggesting that SC administration of tramadol is as effective as IV administration. Increased serum levels of both IL-6 and **β**-endorphin 3 hr postoperatively further indicate that both routes of administration achieve similar pain control. Thus, the relative analgesic efficacy of SC tramadol is comparable to that of IV administration and can be used to achieve similar effects for postsurgical pain management in dogs undergoing OHE.

## 1. Introduction

Inflammatory pain is characterized by an increased sensitivity to mechanical or heat stimuli in the affected tissue [1]. Following tissue injury, an inflammatory response is generated by local macrophages and then amplified by migrating blood cells. Various inflammatory mediators, as well as tissue acidification, act synergistically to induce and maintain pain and hyperalgesia [2]. Regardless of the nature of the event initiating tissue damage, pain usually progresses in a predictable manner and eventually leads to multiple organ failure [3].

Pain recognition in humans is subjective, and pain in animals can be challenging to identify because many species have evolved to mask signs of illness and distress [4]. However, it is the consensus among veterinarians and researchers that predictable alterations in animals' behavior are likely to accompany pain. It is therefore accepted that pain or distress in animals, including those used for biomedical research, should be prevented or minimized as much as possible [5].

In veterinary practice, the clinical application of most nonsteroidal anti-inflammatory drugs for pain management is limited due to their potentially harmful side effects, which include gastrointestinal discomfort, cardiopulmonary depression [6], and decreased antibody production [7]. The use of opioids in the management of both acute and chronic pain is therefore a useful alternative. Opioids are considered the most reliable and effective analgesic, although they can produce reversible behavioral [8] and physiological [9] side effects in dogs.

Tramadol is an analgesic with a dual mechanism of action. It both binds to the *μ*_1_ opioid receptor [10, 11] and inhibits the monoaminergic pathway, which is responsible for noradrenaline (NA) and serotonin (5HT) reuptake [12, 13]. For this reason, tramadol is considered an “atypical opioid” and is only partially inhibited by naloxone, an opioid antagonist [13]. Preemptive administration of tramadol has been shown to significantly reduce the amount of inhalant anesthetic required for procedures in humans [14] and dogs [15].

Several studies have described the efficacy of tramadol following intravenous (IV) [11, 13], oral [11, 16], intramuscular [17] and epidural administration [18]. The subcutaneous (SC) administration of analgesic drugs has been advocated to reduce the risk of overdose and to sustain effective plasma levels for longer durations [19]. Despite the wide use of SC drugs in dogs, the efficacy of SC tramadol for pain control after surgery is not well documented. Although previous studies have investigated the SC administration of tramadol in red-eared sliders [20], chicks [21], and cats [22], none have assessed its efficacy in dogs. This study aimed to compare tramadol's efficacy when administered SC versus IV as a postoperative analgesic agent in dogs undergoing routine ovariohysterectomy (OHE).

## 2. Materials and Methods

Female dogs (*n* = 12) ranging in age from 1 to 3 years old (1.95 ± 0.65) and weighing between 10.5 and 17.1 kg (13.12 ± 1.95) were obtained from a local animal shelter where they were available for adoption (Progressive Animal Welfare Society, PAWS, Subang Airport road, Malaysia). All dogs were determined to be clinically healthy based on a physical examination by a qualified veterinarian prior to surgery. The dogs were familiarized with the handler prior to the study and individually housed in a kennel that was kept quiet and clean. The study was ethically approved by the Universiti Putra Malaysia Animal Care and Utility Committee (UPM/FPV/PS/3.2.1.551/AUP-R86) and adhered to the Declaration of Helsinki.

## 3. Experimental Procedures

Dogs were fasted for 12 hr prior to surgery but had water ad libitum until two hours before premedication. On the morning of surgery, a 20-gauge, 1.25-inch sterile catheter (Terumo, Somerset NJ, USA) was placed and secured in one of the cephalic veins. A baseline venous blood sample (2 mL) was collected from each dog prior to the administration of tramadol. The dogs were randomly divided into 2 groups (*n* = 6 for each group) depending on the route of tramadol administration. At the time of premedication, group 1 received 3 mg/kg tramadol SC, while group 2 received the same dose of tramadol IV.

## 4. Anesthesia and Surgery

The dogs were premedicated with atropine (0.04 mg/kg) (Troy laboratory PTY Ltd, Australia) and xylazine (0.5 mg/kg) (Troy laboratory PTY Ltd, Australia). Anesthesia was induced with ketamine hydrochloride (Narketan, Vetoquinol SA, 70204 Lure, Cedex, France) at 10 mg/kg (IV) given to effect. Following jaw relaxation, the dogs were quickly intubated, and the lungs were ventilated by an assistant using an Ambu bag. The dogs were then prepared for surgery aseptically, and OHE was performed through a ventral midline approach. The same veterinarian performed every surgery, and the mean duration of the procedure was approximately 23 min (22.5 ± 4.6 min).

## 5. Pain Assessment

Pain was assessed both subjectively and objectively. Physiologic parameters, such as cardiopulmonary rates and rectal temperature before and after the surgery, were measured based on criteria defined by the University of Melbourne [23]. In addition, serum levels of interleukin-6 (IL-6) and *β*-endorphin, as well as mechanical pressure thresholds, were measured for the unrestrained dogs before and after surgery.

Pain was evaluated before the surgery as a baseline (0 h) and at 1, 2, 3, 4, 5, 6, 7, and 8 h after recovery from anesthesia. Pain assessments were made by an observer who was blinded with respect to the route of administration. Rectal temperatures and cardiopulmonary rates were recorded for each dog prior to palpation of the surgical site. The mechanical nociceptive thresholds were then measured using a clinical algometer (FPX 25, Wagner instrument, Greenwich CT, USA), which records sensitivity to pressure. Pain was first assessed 2 hr before drug administration and then hourly until 8 hr after anesthetic recovery.

Constant pressure was applied 2 cm from the incision site cranially, caudally, and laterally to the left and right. The pressure threshold was read at the point of a positive reaction, with an average of four readings per site. A positive reaction was considered to be a leg shake, a head turned toward the site, snapping or biting at the instrument, barking, or vocalizing. The instrument was calibrated with a progressively increasing force, and a cut-off point of 15 N was used to prevent mechanical damage to the tissue. Ascending values with an increase in pressure indicated less pain, while descending values with minimal pressure indicated pain in that area.

## 6. Measurement of Serum **β**-Endorphin and IL-6 Levels

Serum *β*-endorphin and IL-6 concentrations were determined before premedication, during surgery (1 hr after tramadol administration), and after surgery at 2, 3, 6, and 9 hr following tramadol administration. Blood samples (2 mL) were collected from a jugular catheter and centrifuged at 1000 ×g for 10 min. The serum was separated and kept frozen at −80°C until analysis with commercially available enzyme-linked immunosorbent assay (ELISA) kits (Cusabio Biotech Co., Ltd, Wuhan China) specific for canine *β*-endorphin and IL-6. A wavelength of 450 nm was selected for optical density measurements on an ELISA microplate reader (Bio-Rad model 680, Bio-Rad Laboratories Inc., Tokyo, Japan).

## 7. Statistical Analysis

Physiological parameters, mechanical pain threshold values, and serum levels of *β*-endorphin and IL-6 were analyzed using parametric statistical methods. Heart rates, respiratory rates, and rectal temperatures were analyzed using repeated measures ANOVA. One-way ANOVA was used to compare differences within treatment groups, and two-way repeated measures ANOVA was used to compare differences between the two treatments over time. Fisher's Least Significant Difference (LSD) multiple comparison test was used to compare differences at each time point. Multiple linear regression analysis was used to test the relationship between objective parameters and the mechanical pain threshold values generated with the analgesiometer. A value of *P* < 0.05 was considered to be significant. The data were reported as the mean ± SD.

## 8. Results

### 8.1. Physiological Parameters

**Figure 1:**
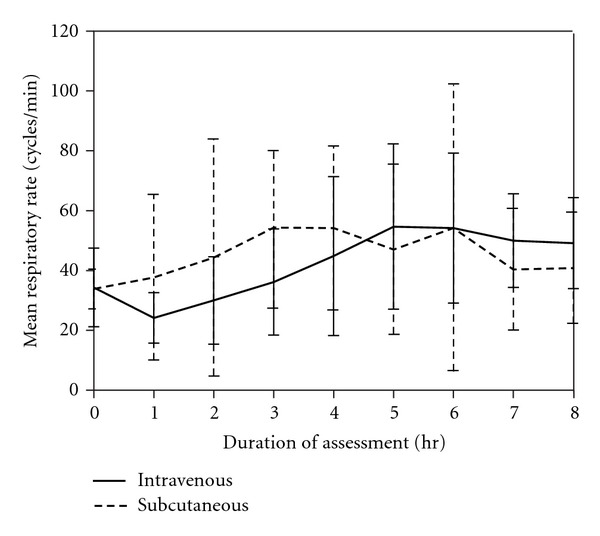
Mean respiratory rates over time for the IV and SC groups (IV group, *P* = 0.069; SC group, *P* = 0.904).

**Figure 2:**
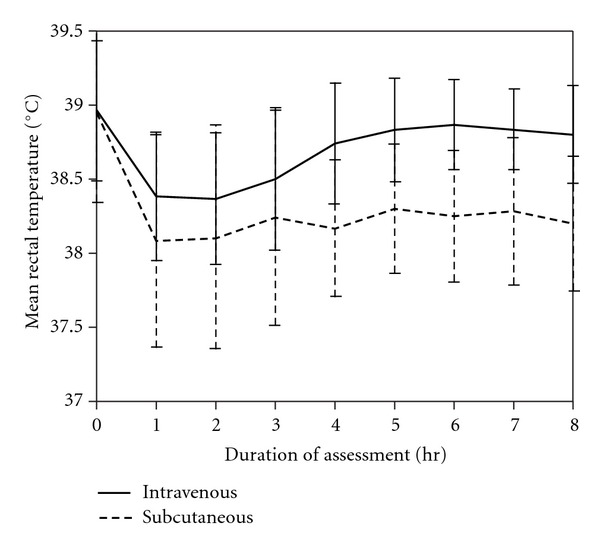
Mean rectal temperatures over time for the IV and SC groups (IV group, *P* = 0.082; SC group, *P* = 0.331).

**Figure 3:**
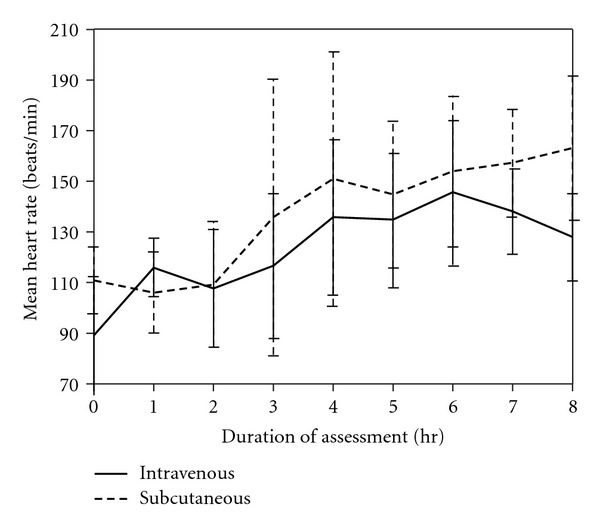
Mean heart rates over time are not significantly different between the IV and SC groups. Values within the IV group are significantly different from the baseline measurements at 4 hr (*P* = 0.02), 5 hr (*P* = 0.02), 6 hr (*P* = 0.00), 7 hr (*P* = 0.001), and 8 hr (*P* = 0.009). Values within the SC group are significantly different from the baseline measurements at 4 hr (*P* = 0.042), 6 hr (*P* = 0.029), 7 hr (*P* = 0.019), and 8 hr (*P* = 0.009).

During the study period, respiratory rate (Figure 1) and rectal temperature (Figure 2) remained within normal range and were not significantly different between or within the two groups, indicating that comparable pain control was achieved. Dogs in both groups had increased heart rates compared to the baseline at 4, 5, 6, 7, and 8 hr (Figure 3), indicating that the dogs recovered at similar rates from anesthesia.

### 8.2. Pain Assessment

No significant differences were observed between the SC and IV groups when they were evaluated for mechanical pain thresholds, indicating similar analgesic effects of tramadol via both routes (Table 1). A significant decrease in the mechanical pain threshold was observed for both groups over time, further indicating that pain perception was similar for the two groups. Respiratory and heart rates were significantly associated with the mechanical pain scores in dogs receiving IV tramadol, but only respiratory rates are significantly associated with the mechanical pain threshold for dogs in the SC group (Table 2).

### 8.3. Serum IL-6 and *β*-Endorphin Levels

**Figure 4:**
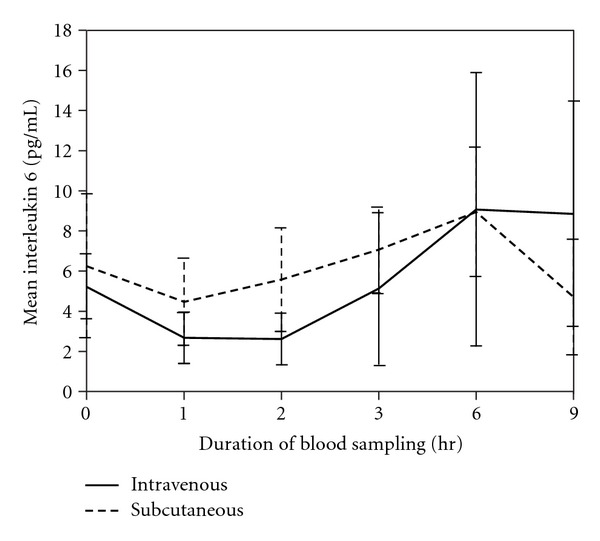
Mean serum IL-6 concentrations for the IV and SC groups at different sampling times after tramadol administration (*P* = 0.701).

**Figure 5:**
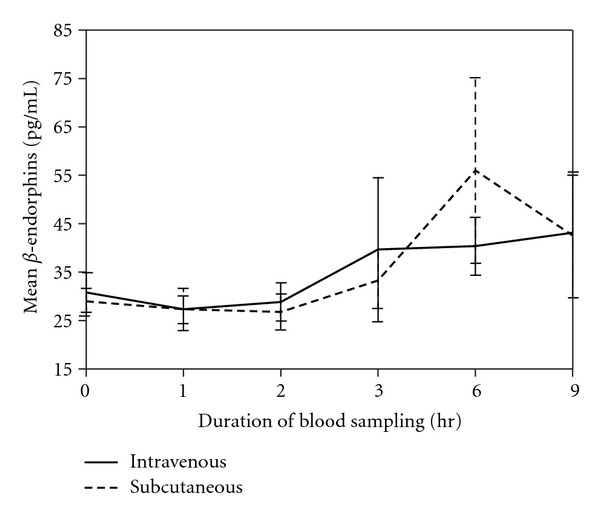
Mean serum *β*-endorphin concentrations for the IV and SC groups at different sampling times after tramadol administration (*P* = 0.806).

There were no significant differences in serum levels of IL-6 (*P* = 0.701) or *β*-endorphin (*P* = 0.806) between the two groups, suggesting that dogs from both groups had similar perception and responses to painful stimulation (Figures 4 and 5).

## 9. Discussion

In humans, SC administration of analgesics is widely used because it achieves relatively similar pain control compared to the intramuscular and IV routes [24]. Information on SC tramadol administration in dogs, however, is limited. Our study is the first to demonstrate that SC administration of tramadol is a reliable and effective method for controlling pain with limited side effects in dogs after OHE.

Although tramadol has a rapid onset of effect when given to dogs IV or intramuscularly, it can also produce unwanted side effects such as nausea, salivation, increased swallowing, and retching [11, 13]. Another effective analgesic strategy, epidural analgesia, carries the risk of nerve injury and side effects that include pruritus, urinary retention, hypoventilation and hypotension, [10, 18, 25]. In contrast, better patient compliance with minimal restraint was achieved in the SC group. In this study, none of the dogs suffered from adverse effects of tramadol, regardless of the route of administration. According to Hendrix et al. [26], marked respiratory depression is not observed in dogs, even with morphine administration. In addition, although vomiting is reported to occur within 5–10 min of SC administration of morphine, this effect is not reported with other opioids [27].

Physiologic indicators of acute pain in animals include increased heart rate, increased blood pressure, peripheral vasoconstriction, cardiac dysrhythmias, sweating, hyperventilation, and reduced peristalsis [27]. In our study, other than the slightly decreased rectal temperatures observed in the SC group two hours after recovery, heart rates, respiratory rates, and rectal temperatures did not vary significantly from the baseline or between the groups. Similar observations were made following epidural administration of tramadol in dogs undergoing stifle surgery [10]. In horses that received epidural tramadol, good analgesia was achieved without significantly influencing the horses' behavioral and physiological parameters [28]. The parameters evaluated here demonstrated that the severity of pain had no detectable physiologic influence on the patients as judged by an observer, and the variability among these parameters was consistent for all tests between the groups. It is important to note that the anesthetic agents used in this study may have resulted in residual central nervous system depression, making pain assessment a challenge postoperatively.

Based on consistently decreasing algometer values for the nociceptive thresholds beginning 2 hours after recovery, increased pain was suspected around the incision site and thus warranted an additional dose of tramadol to maintain analgesia in dogs from both groups. These results suggest that the increased pressure thresholds until 3 hr after tramadol administration are associated with adequate analgesia, although tramadol dosed at 3 mg/kg results in no more than 3 hours of postsurgical analgesia in dogs [11]. The general lack of effect on the pressure threshold after 3 hours may be due to the complexity of pain neurophysiology, the different receptors involved [22], and the rapid clearance of tramadol in dogs [11]. In veterinary medicine, tramadol is typically used in dogs and cats at doses that do not exceed 4.0 mg/kg [29].

In the present study, the subjective pain scales combined with the mechanical nociceptive thresholds indicated that SC tramadol is similar in efficacy to IV in controlling pain after OHE. The effectiveness of the SC route for postsurgical pain management was previously demonstrated in a study comparing the analgesic effects of butorphanol and meloxicam in dogs [30] and tramadol in cats [31] after elective OHE. Progressive increases in the pain scores 3 hours after recovery correspond to the decreasing values observed for the mechanical pain thresholds, suggesting that pain sensation around the incision area increased over time. Due to the rapid elimination of tramadol, a dosing frequency of every four hours is necessary to maintain plasma concentrations sufficient for analgesia in dogs [11, 13]. In humans, the elimination half-life of 6 to 7 hours provides prolonged analgesia and a less frequent dosing schedule [32, 33]. Both subjective and objective indicators for pain were negatively correlated with respiratory and heart rates to mechanical pain thresholds but were positively correlated with rectal temperatures in both groups. Similar relationships were observed in a study that established interval level measurements for a prototype composite measure pain scale assessing acute pain in dogs and in a prospective study that investigated experimental orthopedic pain in beagle dogs [34, 35].

The IL-6 and *β*-endorphin levels may be useful objective indicators of pain in animals [2, 36]. Over the years, numerous studies on the role of cytokines in pain control have been conducted. During pathologic situations associated with increased pain and hyperalgesia, the pleiotropic cytokine IL-6 is markedly upregulated. Elevated serum IL-6 levels were detected in patients with neurological disorders, musculoskeletal injuries, and autoimmune and inflammatory conditions [2]. In the current study, serum IL-6 levels decreased gradually after tramadol administration and remained low throughout the surgery.

A significant increase in IL-6 over the baseline was observed at 3, 6, and 9 hours following administration (*P* = 0.028) via both routes. This observation is similar to that in previous reports, in which gradual increases in IL-6 were observed after both laparoscopic and open surgeries [37]. Similar increases in IL-6 plasma concentrations have been noted in response to cardiopulmonary bypass [38]. Proinflammatory and pronociceptive roles for IL-6 have been described in rats receiving intraplantar and intrathecal injections of IL-6 to induce hyperalgesia or allodynia [39]. In a human study, low synovial fluid concentrations of IL-6 and reduced joint pain were reported following tramadol administration [40], demonstrating its analgesic efficacy and cytokine modulating effects.

*β*-endorphin plays a significant role as biomarker [36] and modulator in acute traumatic conditions such as surgery [41]. Measurements of plasma concentrations of *β*-endorphin have been used extensively in the assessment of pain following castration and tail docking [42]. In our study, serum *β*-endorphin levels at 1 and 2 hours after tramadol administration were significantly lower than levels between 3 and 9 hours after administration. The sharp rise of plasma *β*-endorphin beginning at 3 hours after surgery may be related to the rapid metabolism and clearance of tramadol in dogs [11, 13] and can be correlated with postsurgical pain stimulation. Both groups exhibited similar pain responses as indicated by *β*-endorphin levels. A previous study indicated that serum *β*-endorphin levels over time in severely injured dogs exceeded the levels in moderately traumatized dogs [43]. The increases in serum concentrations due to painful stimuli remained elevated much longer than those due to nonpainful stimuli such as stress from handling [36].

A future crossover study in which dogs receive medication via both routes of administration may help to confirm these findings. In addition, it will be useful to investigate the effects of tramadol administered SC at multiple doses and establish the pharmacokinetic profile of the drug given by this route.

## 10. Conclusion

Postsurgical pain management strategies are improving with the incorporation of SC administration of drugs. The identification of less painful and more reliable routes of drug administration is very important for effectively controlling pain in small animals. We conclude that the relative analgesic efficacy of SC tramadol is comparable to IV administration and can be used independently for postsurgical pain management in dogs. One significant difference between the two routes of administration is the faster time of onset for IV tramadol, which must be considered in emergency situations when a more rapid onset and distribution of analgesia is required.

## Figures and Tables

**Table 1:** Reaction times in the analgesiometer test demonstrate a progressive decrease in the mechanical pain threshold for dogs that received tramadol via both routes, indicating comparable increases in pain perception over time. The results are listed as the mean ± SD.

Time (hr) before (0) tramadol administration and after recovery from anesthesia	Mechanical pain threshold assessment score (Newton)	
	IV group (*n* = 6)	SC group (*n* = 6)
0	14.92 ± 0.1	14.76 ± 0.5
1	15.00 ± 1.8	14.94 ± 1.6
2	14.90 ± 1.6	15.00 ± 0.1
3	7.99 ± 2.7	7.77 ± 2.0
4	5.96 ± 2.8	6.66 ± 2.1
5	4.94 ± 2.2	5.00 ± 1.0
6	4.34 ± 2.1	3.68 ± 0.4
7	3.41 ± 2.0	3.20 ± 0.4
8	2.76 ± 1.8	2.61 ± 0.7

**Table 2:** The relationship between the subjective parameters and mechanical pain threshold was assessed using multiple linear regression.

	IV route				SC route			
Model			Confidence interval				Confidence interval	
Constants independent variables	Beta	*P* value (*P* < 0.05)	Lower bound	Upper bound	Beta	*P* value (*P* < 0.05)	Lower bound	Upper bound

Respiratory rate (cycles/min)	−0.472	0.001	−0.19	−0.053	−0.319	0.008	−0.101	−0.016
Rectal temperature (°C)	0.004	0.972	−3.029	3.139	0.13	0.244	−0.779	2.996
Heart rate (beats/min)	−0.16	0.233	−0.08	0.20	−0.652	0.00	−0.121	−0.059

Dependent variable: mechanical pain threshold (*N*).
